# Real-Time PCR for detection of herpes simplex virus without nucleic acid extraction

**DOI:** 10.1186/1471-2334-6-104

**Published:** 2006-06-24

**Authors:** Mark W Pandori, John Lei, Ernest H Wong, Jeffrey Klausner, Sally Liska

**Affiliations:** 1 San Francisco Department of Public Health, San Francisco, CA 94102, USA

## Abstract

**Background:**

The speed and sensitivity of real-time polymerase chain reaction (PCR) have made it a popular method for the detection of microbiological agents in both research and clinical specimens. For the detection and genotyping of herpes simplex virus (HSV) in clinical specimens, real-time PCR has proven to be faster, more sensitive and safer than earlier methods which included isolation of the virus in cell culture followed by immunofluorescence microscopy. While PCR-based assays for HSV detection posses clear advantages over these earlier techniques, certain aspects of the PCR method remain onerous. The process of extraction and purification of nucleic acid from clinical specimens prior to PCR is particularly cumbersome. Nucleic acid extraction is expensive, time-consuming and provides a step whereby specimens can become contaminated prior to their analysis. Herein, we investigate the necessity of nucleic acid extraction from swab-based clinical specimens for HSV detection by real-time PCR. We find that nucleic acid extraction is unnecessary for specific and sensitive detection of HSV in clinical specimens using real-time PCR.

**Methods:**

Prospective (n = 36) and retrospective (n = 21) clinical specimens from various anatomical sites were analyzed for the presence of herpes simplex virus 1 or 2 by real-time PCR using the RealArt HSV 1/2 LC PCR Kit. Specimens were analyzed by PCR both before and following automated nucleic acid extraction. PCR using extracted and unextracted specimens was also compared to cell culture as a means of detecting HSV.

**Results:**

Detection of HSV 1/2 DNA in clinical specimens by real-time PCR did not require that the specimen be subjected to nucleic acid extraction/purification prior to analysis. Each specimen that was detectable by real-time PCR when analyzed in the extracted form was also detectable when analyzed in the unextracted form using the methods herein. The limit of detection of HSV-1 and HSV-2 particles when analyzed in the unextracted form was found to be approximately 17 and 32 virus particles respectively, compared to a sensitivity of 10 copies, for analysis of purified DNA. Omission of the nucleic acid extraction step shortened both the assay time and cost.

**Conclusion:**

Omission of the nucleic acid extraction step prior to real-time PCR for detection of herpes simplex virus resulted in a more rapid and cost-effective assay, with little impact upon the sensitivity of detection.

## Background

Reliable methods for detection and sub-typing of HSV infections have included enzyme-linked immunosorbent assay (ELISA), immunofluorescence microscopy (IFA) and virus isolation by cell culture. While each of these methods has been very useful in assisting clinical diagnosis, time and technological progress have revealed the limitations of these assays. All three assays are laborious and time consuming, with cell culture often requiring as long as seven days before results are obtained. The sensitivities of these techniques have also been questioned, particularly in reference to more recent methodologies, such as polymerase chain reaction (PCR). The advent of real-time PCR for sensitive and rapid detection of nucleic acid sequences has had a significant impact upon detection of infectious disease agents. Many laboratories, including our own, have adopted real-time PCR as the primary method for detection of HSV due to the speed, sensitivity and relative lack of complexity of the real-time PCR method [1-4]. Typically, specimens analyzed by real-time PCR must first be processed in such a way that nucleic acid is extracted and purified from the clinical specimen. The extracted nucleic acid is used as a reactant in PCR to determine if the DNA sequences of interest (i.e. an infectious agent) are present. Extractions of DNA are deemed necessary due to both assumption and empirical observation that the efficiency of PCR chemistry can be negatively affected by constituents of biological specimens. While it is true that gross contamination of nucleic acid specimens with biological and chemical factors can inhibit PCR, there are few if any reliable trends which describe such inhibition. Polymerase chain reactions require evaluation on a case-by-case basis to determine their efficiency.

Herein, we show that HSV specimens (swabs diluted in a widely-used, commercially available viral transport buffer) are capable of being analyzed by PCR in the absence of any purification or extraction of nucleic acid. Performing PCR on crude specimens does not require any sacrifice of specificity and requires only a minor sacrifice of assay sensitivity.

## Methods

Specimens (n = 36) considered for possible HSV infection were collected from outpatients of the STD clinic during October 2005. Specimens were taken by swabbing of lesions, rashes or ulcers from various anatomical sites, including genital (male and female), rectal (male) and facial (male). Swabs were placed into 2 ml of either Cellmatics or Universal Transport Kit buffer (Becton Dickinson, Sparks, MD). Specimens were refrigerated at 4°C until analyzed, and subsequently frozen at -35°C. Retrospective specimens (n = 21) taken between June 2005 and October 2005 and stored at -35°C were also chosen for analysis. Retrospective specimens were chosen from males (n = 13) and females (n = 8), from various anatomical sites (genital, rectal, facial). Aliquots (1 ml) of all prospective and retrospective specimens were combined with A549 cells (Viromed Laboratories, Minnetonka, MN) in shell vials and placed at 37°C. Cultures were visualized 24, 48, 72 and 168 hours after initiation for determination of the presence or absence of cytopathic effect. If cytopathic effect was noted within a cultured specimen, cells from that culture were harvested and smeared onto glass slides prior to being fixed and subjected to immunofluorescent microscopy using the PathoDx Herpes Typing Kit (Remel, Lenexa, KS) in order to confirm the detection of HSV and to determine HSV type (1 or 2)

For detection of HSV by real-time PCR, samples (200 μl) of each clinical specimen were combined with 200 μl of MagNAPure LC Lysis Buffer and subjected to automated nucleic acid extraction using a MagNAPure LC (Roche Diagnostics, Indianapolis, IN) programmed for Total "Nucleic Acid Extraction Kit I" with external lysis. Final elution volume of each sample at the conclusion of nucleic extraction was 50 μl. Specimens were either analyzed by PCR immediately following extraction, or were stored at -35°C. PCR for the detection of HSV 1/2 DNA was carried out using the RealArt HSV 1/2 LC PCR Kit (Qiagen, Germantown, MD). Reactions were set-up and performed according to manufacturer's instructions. In cases of extracted specimens, 5 μl of extracted sample was added to 15 μl of PCR Master Mix and 0.5 μl of internal control DNA. For unextracted samples, 1 μl of clinical specimen was combined with 4 μl of deionized water, 0.5 μl of internal control DNA and 15 μl of PCR Master Mix. All reactions were performed in a LightCycler 2.0 (Roche, Indianapolis, IN). Data analysis was carried-out using LightCycler 4.0 software, with criteria for positive detection of HSV being designated as any specimen having a crossing point (CP value) less than 30 (using the 640 nm/back 530 nm channel for analysis). This CP value was chosen as follows: Based on our laboratory results, 10 purified HSV-2 DNA copies was found to be detectable 100% of the time (4/4 attempts in one experiment), with the highest CP value being 26.77. Five copies was not detectable (0/4 attempts in two experiments). Results were similar for HSV-1, with 25.53 being the highest CP for detection of 10 purified DNA copies. Hence, for purposes of this work, we set the upper boundary for calling an HSV specimen positive at approximately three crossing points higher than 26.77 (to a CP of 30) to account for the possibility of delays in amplification caused by potential impurities when unextracted clinical specimens are analyzed. Specimens with crossing points greater than 30 were considered negative for HSV. In accordance with the Code of Federal Regulations Title 45 Part 46, this work is exempt from human subjects review as this research involved the study of diagnostic specimens in a manner that patients cannot be identified either directly or through identifiers linked to the specimens.

## Results

### Determining the feasibility of efficient detection of HSV DNA by real-time PCR on untreated clinical specimens

Having established real-time PCR within our laboratory as the method of choice for detection of HSV in clinical specimens, we sought to explore the temporal efficiency of our real-time PCR procedure. We found that approximately 50% (2 hrs.) of the total time required to execute the assay procedure was spent on the process of extraction of nucleic acid from clinical specimens. We investigated whether it would be feasible to detect HSV DNA in crude (unextracted) clinical specimens using the same Real-Time PCR reagents and methods currently utilized in our laboratory. We hypothesized that the diluted nature of the swab specimens that we regularly analyze, along with the typical lack of any gross contamination of the viral transport buffers would allow specimens to be analyzed directly by PCR. Also considered in this hypothesis, was the fact that the first 10 minutes of our PCR procedure included a 95°C denaturation step, which might allow for adequate dissociation of viral nucleic acid from other viral and host components.

**Figure 1:**
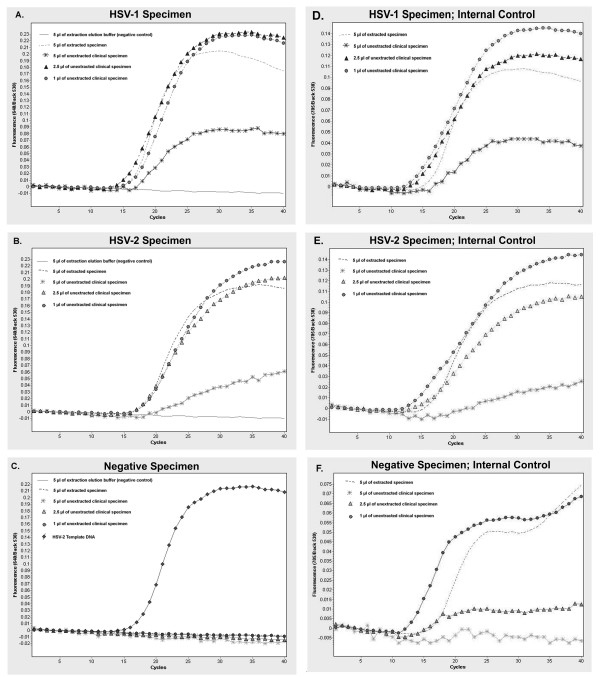
**HSV Clinical Specimens, Analyzed by Real-Time PCR Using Extracted and Unextracted Specimens**. Clinical specimens found to be positive for HSV-1 (A), HSV-2 (B), or found to be negative for HSV (C) when analyzed by cell culture and immunofluorescence microscopy were analyzed by real-time PCR using nucleic acid-extracted and unextracted samples of each specimen. The amplification curves for the internal controls of the reactions shown in A, B and C are shown in D, E and F respectively. For extracted samples, 200 μl of a clinical specimen (in viral transport buffer) was subjected to automated total nucleic acid extraction with an elution volume of 50 μl; 5 μl of the eluted sample was analyzed. For unextracted samples, 5 μl, 2.5 μl and 1 μl of straight clinical specimen were analyzed. The specimens were taken by swab of genital lesion of a male (A, D) or female (B, C, E, F). The swabs were placed in viral transport buffer and stored frozen (-35°C) until analysis. Probes hybridizing to HSV DNA were detected in the 640 nm channel of a Light Cycler 2.0. Probes specific for internal control DNA were detected in the 705 nm channel. The positive real-time PCR control in Figure 1C was 10,000 copies of a purified plasmid containing HSV-2 DNA target fragment (provided by the real-time PCR kit).

We explored the feasibility of real-time PCR for the detection of HSV in unextracted clinical specimens by analyzing three specimens which recently had been detected and typed in our laboratory. The three clinical specimens included a positive HSV-1, positive HSV-2 and negative HSV, (as determined by cell culture and IFA). These three specimens (200 μl each) were subjected to automated nucleic acid extraction with a 50 μl elution volume per specimen. Extracted specimens (5 μl) were then analyzed by HSV 1/2- specific real-time PCR. Simultaneous to this, samples of those same three clinical specimens were also analyzed by real-time PCR, using 5, 2.5 and 1 μl of crude, unextracted specimen combined with water (if necessary) to achieve a final volume of 5 μl. As shown in Figure 1, the amplification curves for 5 μl of extracted specimens were nearly identical to the curves generated when either 1 μl or 2.5 μl of crude specimen was analyzed. This finding was true for both the HSV-1 and HSV-2 specimens tested (Figures 1A, 1B). The use of 5 μl of unextracted clinical specimen did not appear to significantly alter the crossing points of either specimen relative to extracted sample. However, the use of 5 μl of unextracted specimen did have an impact on some aspect of the amplification or detection process, as such curves possessed jagged compositions, with much lower maximum fluorescence. Known negative clinical specimen did not show any amplification when 5, 2.5 or 1 μl of crude, unextracted specimen were subjected to PCR relative to the extracted version of the same specimen (Figure 1C).

Included in all real-time PCR reactions was an internal control which utilized the same primers, but a different probe than those used to detect HSV (the probes for these internal controls emit light at a wavelength of 705 nm) (Figures 1D, 1E and 1F). The internal control reactions functioned properly (i.e. they showed exponential amplification) for all specimens shown in figures 1A, 1B and 1C when either 1 or 2.5 μl of specimen was analyzed. However, in each case where 5 μl of crude clinical sample was analyzed, the internal controls either failed to amplify efficiently (Figures 1D, 1E), or did not amplify at all (Figure 1F) indicating that the fundamental chemistry of PCR was negatively affected by something in the crude specimen, but that a significant amount of the crude specimen was required to be added to the reaction for such a negative impact to occur. In the cases where HSV-1 and HSV-2 clinical specimens were tested (Figures 1D and 1E), amplification of the internal control was merely delayed when 5 μl of clinical specimen was used. Hence, these specimens would have still been considered as valid specimens for analysis by the testing protocol utilized herein. However, in the case of the negative clinical specimen in which 5 μl of raw specimen was tested (Figure 1F), amplification of the internal control was completely inhibited. Such a specimen would not have been considered 'negative' for HSV. Rather, this specimen would have been considered invalid, and an additional clinical specimen from the patient would have to have been ordered.

### Evaluation of PCR performance with extracted and unextracted prospective and retrospective specimens

With preliminary evidence that crude, unextracted clinical swab specimens, when used in the proper amounts, are adequate for direct PCR analysis, we sought to determine the repeatability of this finding. Consecutive prospective specimens submitted to our laboratory for HSV testing (n = 36) were subjected to attempted isolation by cell culture. All cultures with evidence of cytopathic effect were subsequently tested by immunofluorescence assay (IFA) for typing. Simultaneous to those tests, specimens were subjected to HSV-specific real-time PCR in either extracted (5 μl) or unextracted (1 μl) form. Although both 1 μl and 2.5 μl of crude specimen had performed adequately in PCR in our initial feasibility study, as shown in Figures 1A and 1B, we chose to use 1 μl for the remainder of the study for the reason that such a volume would carry over a smaller amount of potentially inhibiting factors, if any, in the clinical specimen. As shown in Table 1 [see Additional File 1], using a crossing point of 30 as the limit of detection, the ability to detect HSV DNA(either type 1 or type 2) in clinical specimens was perfectly concordant for extracted and unextracted specimens. The genetic typing of positive specimens as either HSV-1 or HSV-2 was also 100% concordant between extracted and unextracted specimens. One specimen determined to be undetectable by the method of viral culture was found to be positive by PCR whether the reaction was performed on extracted or crude samples of that specimen. These findings reinforce that nucleic extractions are not necessary when analyzing clinical specimens for the presence of HSV DNA by PCR. These data also indicate that PCR can still be a more sensitive method than viral culture as a means of HSV detection, whether or not the tested specimen is subjected to nucleic acid extraction prior to analysis. All specimens found to be negative by real-time PCR possessed exponential amplification curves for internal control PCR (data not shown).

**Table 1:** Prospective Clinical HSV Specimens

**Specimen**		**Culture/IFA**		**Real-Time PCR**					
				**Extracted^a^**			**Unextracted^b^**		

Gender	Site	Culture	IFA	Result	CP value	Type	Result	CP value	Type

F	genital	positive	HSV-2	positive	9.87	HSV-2	positive	12.49	HSV-2
M	genital	positive	HSV-2	positive	10.95	HSV-2	positive	12.16	HSV-2
M	genital	positive	HSV-1	positive	11.03	HSV-1	positive	12.18	HSV-1
F	genital	positive	HSV-2	positive	11.82	HSV-2	positive	12.70	HSV-2
F	genital	positive	HSV-2	positive	13.10	HSV-2	positive	15.28	HSV-2
M	genital	positive	HSV-2	positive	13.35	HSV-2	positive	15.69	HSV-2
F	genital	positive	HSV-2	positive	13.80	HSV-2	positive	16.83	HSV-2
F	genital	positive	HSV-2	positive	13.97	HSV-2	positive	15.20	HSV-2
M	genital	positive	HSV-1	positive	15.27	HSV-1	positive	16.09	HSV-1
M	genital	negative^c^	n/a	positive	16.06	HSV-1	positive	18.18	HSV-1
F	genital	positive	HSV-2	positive	16.97	HSV-2	positive	17.22	HSV-2
M	genital	positive	HSV-2	positive	17.47	HSV-2	positive	18.85	HSV-2
F	genital	positive	HSV-2	positive	17.60	HSV-2	positive	18.49	HSV-2
M	rectal	positive	HSV-2	positive	19.48	HSV-2	positive	21.82	HSV-2
M	genital	positive	HSV-1	positive	20.37	HSV-1	positive	22.29	HSV-1
M	genital	positive	HSV-2	positive	20.66	HSV-2	positive	22.60	HSV-2
F	genital	positive	HSV-2	positive	22.64	HSV-2	positive	23.92	HSV-2
F	genital	negative	n/a	negative		negative	negative		negative
M	rectal	negative	n/a	negative		negative	negative		negative
M	rectal	negative	n/a	negative		negative	negative		negative
F	rectal	negative	n/a	negative		negative	negative		negative
M	rash/lesion	negative	n/a	negative		negative	negative		negative
M	genital	negative	n/a	negative		negative	negative		negative
F	genital	negative	n/a	negative		negative	negative		negative
M	rectal	negative	n/a	negative		negative	negative		negative
M	genital	negative	n/a	negative		negative	negative		negative
M	genital	negative	n/a	negative		negative	negative		negative
F	genital	negative	n/a	negative		negative	negative		negative
M	rectal	negative	n/a	negative		negative	negative		negative
M	genital	negative	n/a	negative		negative	negative		negative
M	genital	negative	n/a	negative		negative	negative		negative
M	rash/lesion	negative	n/a	negative		negative	negative		negative
M	rectal	negative	n/a	negative		negative	negative		negative

^a ^5 ul of extracted nucleic acid used in PCR

^b ^1 ul of straight clinical specimen used in PCR

^c ^specimen was negative for HSV by cell culture; positive by real-time PCR

n/a specimen was not analyzed

CP value Crossing Point of real-time PCR

To determine whether the physiological source of the clinical specimen affects whether extraction is necessary for PCR analysis, we extended our analysis to include 21 retrospectively evaluated specimens. Specimens were selected so that a range of samples from various anatomical sites, from both sexes, would be represented. In addition, one of the chosen retrospective specimens was selected because it had previously been found to be negative by cell culture but positive by PCR (using extraction) in our laboratory. This specimen was analyzed in order to determine whether the improved sensitivity demonstrated by PCR over cell culture using extracted specimens could be maintained when the PCR was performed using unextracted specimens.

As shown in Table 2 [see Additional File 2], results for PCR testing of extracted and unextracted versions of all retrospective specimens indicated that both forms of specimens were detectable. One specimen which was previously determined to be negative by way of cell culture and positive by real-time PCR was found to be positive by PCR whether or not the specimen was subjected to extraction. All specimens found to be negative by real-time PCR possessed exponential amplification curves for internal control PCR (data not shown). These data confirm that extraction of nucleic acid from clinical HSV specimens is not necessary prior to PCR detection, and that the enhanced sensitivity of PCR over cell culture for HSV detection is at least not completely sacrificed when the extraction step is bypassed. Moreover, these data indicate that clinical specimens taken from a variety of anatomical sites may be subjected to PCR without prior nucleic acid extraction.

**Table 2:** Retrospective Clinical HSV specimens

**Specimen**		**Culture/IFA**		**Real-Time PCR**					
				**Extracted^a^**			**Unextracted^b^**		

Gender	Site	Culture	IFA	Result	CP value	Type	Result	CP value	Type

M	rectal	positive	HSV-1	positive	7.09	HSV-1	positive	10.44	HSV-1
F	genital	positive	HSV-1	positive	8.49	HSV-1	positive	11.01	HSV-1
F	genital	positive	HSV-2	positive	12.55	HSV-2	positive	15.28	HSV-2
F	genital	positive	HSV-2	positive	13.14	HSV-2	positive	15.32	HSV-2
M	genital	positive	HSV-1	positive	13.14	HSV-1	positive	14.98	HSV-1
M	genital	positive	HSV-1	positive	13.23	HSV-1	positive	15.68	HSV-1
M	rectal	positive	HSV-1	positive	13.27	HSV-1	positive	14.51	HSV-1
M	genital	positive	HSV-2	positive	13.70	HSV-2	positive	15.98	HSV-2
M	rash/lesion	positive	HSV-1	positive	13.73	HSV-1	positive	15.82	HSV-1
F	genital	positive	HSV-1	positive	14.54	HSV-1	positive	15.53	HSV-1
F	rash/lesion	positive	HSV-2	positive	14.87	HSV-2	positive	17.99	HSV-2
M	rectal	positive	HSV-2	positive	15.84	HSV-2	positive	20.13	HSV-2
F	genital	negative^c^	n/a	positive	17.91	HSV-2	positive	19.88	HSV-2
M	rash/lesion	positive	HSV-2	positive	18.35	HSV-2	positive	21.31	HSV-2
M	rectal	positive	HSV-2	positive	19.96	HSV-2	positive	21.58	HSV-2
F	rash/lesion	positive	HSV-1	positive	20.73	HSV-1	positive	22.36	HSV-1
M	genital	positive	HSV-2	positive	23.56	HSV-2	positive	24.98	HSV-2
M	genital	negative	n/a	negative		negative	negative		negative
M	rash/lesion	negative	n/a	negative		negative	negative		negative
F	genital	negative	n/a	negative		negative	negative		negative
M	rectal	negative	n/a	negative		negative	negative		negative

^a ^5 ul of extracted nucleic acid used in PCR

^b ^1 ul of straight clinical specimen used in PCR

^c ^specimen was negative for HSV by cell culture; positive by real-time PCR

n/a specimen was not analyzed

CP value Crossing Point of real-time PCR

### Comparison of the sensitivities of PCR using extracted and unextracted specimens

On a qualitative basis, the data in Tables 1 and 2 [see Additional Files 1 and 2] show 100% concordance of PCR results for extracted and unextracted clinical specimens. However, inspection of the crossing point values of each analyzed specimen reveals a trend of disparity between extracted and un-extracted specimens. For prospectively analyzed HSV-2 specimens, the average crossing point for extracted specimens was 14.69, while the average crossing point for the same specimens analyzed in unextracted form was 16.26 (a difference of 1.57). Similarly for prospective HSV-1 specimens, the averages for extracted and unextracted specimens were 15.68 and 17.17 respectively (a difference of 1.49). These differences indicate that specimens analyzed in the extracted form are more readily detected than specimens run in unextracted form. Such a difference would be expected, based on the methodology: During nucleic acid extraction, 200 μl of clinical specimen is lysed, purified, and finally eluted in 50 μl of elution buffer. Hence, assuming that nucleic acid extraction resulted in 100% recovery of HSV DNA, the eluted specimen theoretically contains 4 μl equivalents of original clinical specimen (200 μl original specimen/50 μl eluted specimen) per microliter. When 5 μl of extracted, eluted sample is analyzed by PCR, this correlates to 20 μl equivalent of original clinical specimen. In this study, when the same clinical specimen was analyzed in unextracted form, only 1 μl of original clinical specimen was utilized. This difference in the amount of extracted and unextracted specimen used in PCR implied that the theoretical sensitivity enhancement of HSV PCR using extracted versus unextracted specimens should be at least 20-fold. Others have found however, that the process of automated nucleic acid extraction we utilized herein results in far less than 100% recovery of nucleic acid from specimens [5]. Hence, we sought to determine the yield of nucleic acid extraction by this automated method in our lab. To do this, we utilized quantified plasmids containing HSV DNA target fragments (provided by the real-time PCR kit), combined with known negative clinical HSV specimens. Such spiked formulations containing known amounts of HSV DNA target fragment were then subjected to nucleic acid extraction, and the eluted samples were quantified by real-time PCR. HSV-2 DNA target fragments (90,000 copies in 200 μl of non-HSV-containing (negative) clinical specimen) were subjected to automated extraction, and were eluted to a final volume of 50 μl. Subsequent quantitative PCR analysis showed that the average yield (recovery) of three extractions was 24.1%. Using this factor, a comparison of sensitivities of the use of extracted and unextracted specimens in PCR was reconsidered: In the context of this work, using 5 μl of extracted specimen was actually the equivalent of analyzing only 4.8 μl of original specimen. Hence, the sensitivity of PCR for HSV detection using extracted specimens is approximately only 5 fold greater than the sensitivity of the same PCR using unextracted specimen. In accordance with this, we have routinely found with the RealArt HSV 1/2 LC PCR Kit that a 3-cycle crossing point differential correlates to an approximate 10-fold difference in target DNA concentration. This in agreement with the finding that extracted specimens possess crossing points approximately 1.5 cycles lesser than those of their unextracted counterparts.

To further evaluate the sensitivity of PCR on unextracted clinical specimens, we performed real-time PCR analysis on dilutions of previously quantified stocks of patient-derived HSV particles. HSV-1 and HSV-2 stocks containing 3.4 × 10^7 ^and 1.02 × 10^8 ^virus particles per millilitre respectively were subject to 2-fold serial dilution using a HSV-negative clinical specimen as a diluent. Dilutions were subject to real-time PCR in the unextracted form to determine the maximum dilution of whole, unextracted virus particles detectable by the assay. For HSV-1, a diluted sample theoretically containing 17 virus particles (1 μl of a 1/2000 dilution) was the maximum detectable dilution, giving a crossing point value of 27.98. For HSV-2, a diluted sample theoretically containing 32 virus particles per microliter (1 μl of a 1/3200) was the maximum detectable dilution, giving a crossing point value of 26.59. Purified HSV-1 and HSV-2 target DNA standard (provided by the kit) were found to be detectable at a minimum level of 10 copies in 4 out of 4 reactions, with the highest detected crossing point values being 26.77 for HSV-2 and 25.53 for HSV-1. These data confirm that there is a sensitivity loss for real-time PCR when HSV is detected in the unextracted form compared to the detection of purified DNA.

## Discussion

The data described in this work indicate that real-time PCR for the detection of herpes simplex viruses can be performed without previous extraction and purification of nucleic acid from clinical samples. It is important to emphasize that this assertion is made only in the context of specimens collected by swabs which have been put into contact with anatomical sites and subsequently diluted into a commonly utilized viral transport buffer. Specimens collected in this way probably contain very little physiological debris, and what little debris that is carried by the swab is diluted greatly into transport buffer. Also, the first step of this (and many) PCR protocol involves 10 minutes at 95°C. At this temperature, many bio-molecules will be denatured and solubilised, allowing for lipids and protein complexes to disassociate, hence allowing for exposure of target nucleic acids to the detection chemistry reactants (e.g. primers, probes, enzymes). Moreover PCR protocols call for a relatively small amount of such diluted specimen be placed within the PCR reaction, further diluting-out any potential inhibitors. Whether or not PCR can be carried out on non-extracted, non-purified specimens is certainly a matter to be determined empirically, on a case-by-case basis. However the data provided herein imply that it may very well be worth considering omission of nucleic acid extraction steps in cases where dilution of potential inhibitors takes place during specimen collection or in cases where the clinically collected specimen is thought to be relatively free of potential inhibitors. Certain conditions demand that nucleic acid extraction and purification be performed prior to PCR. This is true for protocols involving RNA viruses, where reverse transcription of RNA into DNA must take place prior to PCR. Since such protocols involve reverse transcription steps that take place at relatively lower temperatures (often 48°C) before the denaturation step (95°C or greater), access of enzymes to viral RNA is required. Attempts to perform reverse-transcription-PCR on specimens known to contain influenza A virus (an RNA virus) in our laboratory were not successful without first extracting and purifying nucleic acid.

Our use of PCR to detect HSV in specimens in lieu of nucleic acid extraction was not without some sacrifice in the sensitivity of the assay. Using 1 μl of raw clinical specimen resulted in an approximate 2 to 5-fold reduction in sensitivity relative to when extracted specimens were used. However, this level of sensitivity loss does not seem considerable in terms of detecting infection in patients. It is typical for skin lesions caused by HSV to secrete very high concentrations of virus particles [1]. This is corroborated by the study herein involving both prospective and retrospective clinical specimens. In all of the positive clinical specimens detected, the highest crossing point value identified by PCR of unextracted specimens was 24.98, with the vast majority of positive specimens possessing crossing point values less than 22, which correlates to approximately 500 virus particles per microliter in our assay (data not shown). Hence, it appears that the consequential loss of sensitivity of approximately 1.5 crossing point values for analysis of unextracted specimens will very rarely render a positive specimen undetectable by the unextracted real-time PCR method. In this body of data, we identified two low-positive specimens (culture-negative, PCR-positive using extracted specimen samples) that were readily detected by real-time PCR when raw, unextracted specimen samples were analyzed. It should also be noted that in this study, we chose to analyze only 1 μl of clinical specimen. This amount was chosen in the interest of generating a conservative estimate of the capabilities of unextracted PCR for HSV, while additionally maintaining a low probability of carrying over a physiologic inhibitor to PCR. Our results showed that as much as 2.5 μl could be analyzed per reaction. If such an amount was used, then the sensitivity difference between PCR on extracted and unextracted specimens might be less than 2-fold. Moreover, the data herein indicate that the use of a crossing point value of 30 as a cut-off for detection of impure specimens may have been unnecessarily high. Noting that the highest crossing point generated for an unextracted specimen was 27.98 for the smallest detected amount of whole virus (17 HSV-1 particles), we intend to consider 28 as a crossing point maximum for assigning positive status to a specimen.

The use of PCR without extensive nucleic acid extraction of specimens is not without precedent. Polymerase chain reaction for the detection of Bordetella pertussis has been reliably achieved using swabs merely agitated in water and heated for ten minutes [6]. Real-time PCR for the detection and quantification of adeno-associated viral vectors was shown to work very well on a routine basis, with a two-fold approximate loss in sensitivity in the ability to detect unextracted AAV particles relative to those subjected to nucleic acid extraction [7].

Other simplifications of the overall process of using PCR to diagnose HSV infections have been shown to be effective. Filen et al have shown that dry cotton swabs in an empty transport tube are just as effective as those placed in viral transport buffer when used subsequently in real-time PCR [1]. Such findings, combined with those in this work, may operate well together towards establishment of a greatly simplified diagnostic protocol. Simplification of the overall protocol might greatly eliminate the possibility of contamination during sample processing, while shortening assay time.

Real-time, quantitative PCR is becoming commonplace in both the clinical and public health laboratory settings. The cost of consumables, reagents and labor that is required to operate a PCR-capable laboratory can be prohibitive. Hence, studies that critically evaluate the relevancy of the individual steps of complex protocols such as PCR may result in modifications to protocols which save time and money, with a minimal sacrifice in assay performance.

## Conclusion

Clinical specimens consisting of swabbed lesions thought to be caused by herpes simplex virus (HSV, type 1 or 2) can be analyzed by real-time PCR in lieu of nucleic acid extraction and purification. Analysis of specimens by real-time PCR without previous extraction/purification of nucleic acid results in an approximate 2 to 5-fold loss in sensitivity, with no discernable loss of specificity. The sensitivity loss which occurs when specimens are analyzed in this way still results in a highly sensitive assay relative to cell culture based isolation. Moreover, the exclusion of nucleic acid extraction results in a considerable savings in both time and money.

## Competing interests

The author(s) declare that they have no competing interests.

## Authors' contributions

MWP designed the experiments, carried out some of the experimentation, analyzed the data and authored the manuscript. JL and EHW carried out experiments. JDK collected clinical specimens and along with SL, provided oversight to the project.

## Pre-publication history

The pre-publication history for this paper can be accessed here:



## Supplementary Material

Additional File 1 Prospective Clinical Specimens. This file is a table with data comparing the techniques of cell culture, immunofluorescent antibody and real-time PCR with and without nucleic acid extraction for the detection of HSV in prospective clinical specimens. Real-time PCR crossing points are shown. Click here for file

Additional File 2 Retrospective Clinical HSV Specimens. This file is a table with data comparing the techniques of cell culture, immunofluorescent antibody and real-time PCR with and without nucleic acid extraction for the detection of HSV in retrospective clinical specimens. Real-time PCR crossing points are shown. Click here for file

## References

[B1] Filén F, Strand A, Allard A, Blomberg J, Herrmann B (2004). Duplex Real-Time Polymerase Chain Reaction Assay for Detection and Quantification of Herpes Simplex Virus Type 1 and Herpes Simplex Virus Type 2 in Genital and Cutaneous Lesions. Sexually Transmitted Diseases.

[B2] Koenig M, Reynolds KS, Aldous W, Hickman M (2001). Comparison of Light-Cycler PCR, enzyme immunoassay, and tissue culture for detection of herpes simplex virus. Diagnostic Microbiology and Infectious Disease.

[B3] Espy M, Uhl J, Mitchell P, Thorvilson J, Svien K, Wold A, Smith T (2000). Diagnosis of Herpes Simplex Virus Infections in the Clinical Laboratory by LightCycler PCR. Journal of Clinical Microbiology.

[B4] Whiley DM, Syrmis MW, Mackay IM, Sloots TP (2003). Preliminary comparison of three LightCycler PCR assays for the detection of herpes simplex virus in swab specimens. Eur J Clin Microbiol Infect Dis.

[B5] Schuurman T, van Breda A, de Boer R, Kooistra-Smid M, Beld M, Savelkoul P, Boom R (2005). Reduced PCR sensitivity Due to Impaired DNA Recovery with the MagNA Pure LC Total Nucleic Acid Isolation Kit. Journal of Clinical Microbiology.

[B6] Lind-Brandberg L, Welinder-Olsson C, Lagergård T, Tranger J, Trollfors B, Zackrisson G (1998). Evaluation of PCR for Diagnosis of Bordetella pertussis and Bordetella parapertussis Infections. Journal of Clinical Microbiology.

[B7] Veldwijk M, Topaly J, Laufs S, Hengge U, Wenz F, Zeller W, Fruehauf S (2002). Development and Optimization of a Real-Time Quantitative PCR-based Method for the Titration of AAV-2 Vector Stocks. Molecular Therapy.

